# Advances in Vulvar Cancer: A Radiation Oncology Perspective

**DOI:** 10.3390/cancers17152415

**Published:** 2025-07-22

**Authors:** Diandra N. Ayala-Peacock, Manjeet Chadha

**Affiliations:** 1 Duke University Medical Center Department of Radiation Oncology, Durham, NC 27710, USA; 2 Department of Radiation Oncology, Ichan School of Medicine at Mount Sinai, New York, NY 10029, USA; manjeet.chadha@mountsinai.org

**Keywords:** vulvar cancer, radiotherapy, chemotherapy, therapeutic approaches, molecular subtyping

## Abstract

Despite being a rare malignancy, there have been several changes in the management paradigm for vulvar cancer. This review of the literature was undertaken to highlight key areas of treatment innovation and progress, including efforts to de-escalate morbid surgical resection as well as perform dose escalation of radiotherapy and incorporation of modern systemic agents to achieve better oncologic outcomes. There is still much debate regarding key high-risk pathology features and their corresponding prognostic significance and indications for adjuvant treatments. However, we are also developing a more nuanced understanding of the importance of precursor lesions and resultant subtypes of vulvar cancer, which suggests that there are more subtypes beyond the umbrella distinction of HPV status. Moving forward, we anticipate there will be an increasing number of trials investigating the triaging of management recommendations based on risk.

## Simple Summary

Vulvar cancer is a rare malignancy, with much of its management extrapolated from more prevalent malignancies such as cervix cancer. Over the last several decades, there has been considerable evolution in both the surgical management and the application of adjuvant therapies for resectable disease, as well as advancements in chemotherapy and radiation for the nonsurgical management of locally advanced vulvar cancer. Novel investigations into the molecular subtypes of vulvar cancer are now beginning to influence management and usher in an era of risk assessment and risk-adapted therapies. This review serves to highlight the current practices and areas of investigation in vulvar cancer management.

## 1. Introduction

Vulvar cancer is a rare malignancy, representing 0.4% to 0.7% of all cancers in US women [1], with 2025 American Cancer Society estimates projecting 7480 new diagnoses and approximately 1770 deaths [2].

Clinically, there are two established pathways of carcinogenesis for this disease— human papillomavirus (HPV)-associated disease or HPV-independent vulvar dystrophy—with each portending distinct phenotypic patient and prognostic differences.

HPV-mediated vulvar cancer is associated with HSIL or usual VIN [uVIN] and often occurs in younger women. HPV is a recognized trigger for basaloid squamous cell carcinomas of the vulva, with HPV DNA being identified in approximately 40% of invasive vulvar cancers and serotypes 16, 18, and 33 being the predominant HPV subtypes [3]. HPV in this disease site follows the pattern of other HPV-mediated malignancies and is felt to be a better prognostic feature [4,5], with several retrospective series demonstrating improved clinical outcomes, including reduced local regional recurrence rates and improved disease-free survival (DFS) [5,6], progression-free survival (PFS), and overall survival (OS). In contrast, vulvar dysplasia such as Lichen sclerosus (LS) is often seen in older women and is associated with HPV-negative keratinizing squamous cell carcinomas [7]. Risk increases with older age and concurrently differentiated VIN [dVIN] with estimates of up to 19% risk of invasive cancer at 10 years [8] [Table 1].

**Figure 1:**
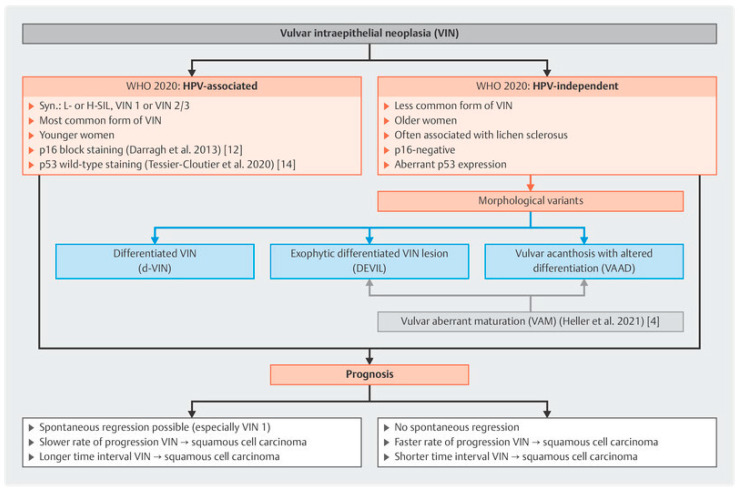
Classification of precursor vulvar intraepithelial lesions and corresponding prognostic significance. Reprinted from AK Höhn, CE Brambs, GGR Hiller, D May, E Schmoeckel and L-C Horn. 2020 WHO Classification of Female Genital Tumors. Geburtsh Frauenheilk 2021; 81: 1145–1153 DOI 10.1055/a-1545-4279) [13].

The WHO recognizes more than one HPV-independent process with at least two known types of HPV-negative VIN. Specifically, there is the differentiated type (dVIN) associated with TP53 alterations in the setting of chronic inflammatory dermatoses, and dVIN p53 WT precursors under the names of differentiated exophytic vulvar intraepithelial lesion (DEVIL) and vulvar acanthosis with altered differentiation (VAAD) [9,10]. Together DEVIL, VAAD, and similar HPV-independent processes that combine aberrant maturation with minimal nuclear atypia, may be referred to as Vulvar Aberrant Mutations (VAM) [11]. [Figure 1]. These HPV-independent p53 WT lesions are associated with invasive squamous cell carcinoma and verrucous carcinoma of the vulva and have been determined to harbor recurrent alteration in oncogenes like PIK3CA, HRAS, and NOTCH1 [9]. In 2020 the WHO Classification proposed the terminology VaVIN as a unifying term for HPV-independent p53WT verruciform acanthotic vulvar intraepithelial neoplasia, with the goal of acknowledging their neoplastic potential [9,12]. Currently these precursor classifications do not impact the clinical management of this rare malignancy but serve as prognostic factors to predict disease behavior and response to therapy.

In an effort to unite biologic markers with therapy management, the Vulvar Immunohistochemical Panel (VIP) Project investigated the immunohistochemical (IHC) expression of 14 biological markers as potential prognostic and therapeutic markers in vulvar SCC. The panel included p16, p53, MLH1, MSH2, MSH6, PMS2, PD-L1, CD3, HER2/neu, ER, PR, EGFR, VEGF, and CD31 and was applied to the tissue from two cohorts of node-negative and node-positive surgically managed patients. They observed a significantly higher p16 expression in the node-negative group (20.8% vs. 6.2%), correlating with the relationship between p16/HPV status and better prognostic outcomes. PD-L1 (Programmed Cell Death Ligand 1) positivity and higher EGFR (Epidermal Growth Factor Receptor) expression were found in the node-positive cohort (77.1% and 97.9% respectively) and overall p16-negative tumors demonstrated a higher PD-L1 expression (60.9% vs. 50.0%) [14]. Mutated p53 and the over-expression of PD-L1 showed significant association with nodal metastasis. All mismatch repair (MMR) proteins showed the retention of the expression, and ER, PR, and HER2/neu were negative and as such, were excluded from further analysis due to the lack of signal/diversity in those stains. Only 14/101 (13.9%) tumors showed a strong–moderate diffuse expression of VEGF (Vascular Endothelial Growth Factor) without differences between the node-negative and node-positive cohorts. The VIP Project did not observe a statistically significant association between high EGFR IHC expression, patients’ survival, and nodal metastases, but recognized controversies in the literature with other series suggesting a possible worse disease-free survival (DFS) in patients [15] and/or decreased survival [16] with high IHC EGFR expression. Still others have suggested an association of EGFR expression with nodal disease without demonstrating ability to predict the presence of nodal disease [17].

The cluster analysis identified three subgroups in the node-negative cohort and four sub-groups of molecular profiles in the node-positive cohort, with no difference in prognosis. Interestingly, the molecular signature of each sampled node and corresponding tumor diverged significantly in 18/41 (43.9%) cases.

Using fluorescence in situ hybridization (FISH), colleagues from Brigham and Women’s used probes targeting CD274 (encoding PD-L1), PDCD1LG2 (encoding PDL2), and the centromeric portion of chromosome 9 paired with IHC for PDL1 signal to evaluate a genetic basis for PD-L1 expression in a subset of cervical and vulvar SCCs [18]. They observed cogain or coamplification of CD274 and PDCD1LG2 in 32 of 48 cervical SCCs (67%) and 10 of 23 vulvar SCCs (43%). Median PD-L1 protein expression was highest among tumors with CD274 and PDCD1LG2 coamplification and lowest among tumors with disomy.

The current NCCN guidelines [19] for management of this rare malignancy are largely based on clinicopathologic features such as tumor size, depth of invasion (DOI), surgical margin distance, and presence or absence of lymphovascular space invasion (LVSI). Efforts to advance management of vulvar cancer have largely focused on de-escalation of extent of surgery for the primary tumor and regional lymph nodes [20], advances in radiation technology and treatment delivery [21,22], and use of multidisciplinary combined modality approach for treating locally advanced disease [23]. Emerging data continues to build on a differential prognosis defined by HPV-independent/TP53-mutant VSCC, having the worst outcomes, and HPV-independent/TP53-wild-type VSCC, with an intermediate prognosis. There is a need to critically review our understanding of molecular characteristics and biology of VSCC with implications for triaging utilization of multimodality therapies to improve clinical outcomes.

## 2. Surgically Resectable Cancer of the Vulva

When possible, the primary treatment modality for vulvar cancer is surgical resection. Contemporary surgical management of early-stage unifocal vulvar cancers has evolved considerably from radical vulvectomy (initially as part of an en bloc resection) to more modern approaches of wide local excision (WLE) or radical local excision with extension to the deep perineal fascia. In 1993 Hacker et al. [24] reported comparable single-digit recurrence rates of around 7% following radical vulvectomy versus WLE, confirming that for early-stage lesions local control could be achieved with smaller surgeries. Furthermore, more modern prospective trials have incorporated WLE without any evidence to suggest higher failure rates in this population [20].

Transitioning from en bloc resections to separate surgical incision approaches allowed for decoupling of the resection of the primary along with resection of the inguinal and often pelvic lymph nodes, with lower surgical complication rates. However, despite the reduction in morbidity with separate surgical incision approaches, this procedure continues to result in persistently high morbidity rates of wound breakdown and infection (20–40%) as well as lymphedema ranging from 30–70% [25,26,27]. More recently sentinel-node procedure (SLNBx) in early stage (unifocal < 4 cm) vulvar cancer management has gained wide acceptance as a safe and validated alternative to IFND for patients with early-stage disease (T1 or T2 < 4 cm).

Through a series of prospective single arm study design the GROINS V, GROINSS V I, and GROINSS V II trials have been informative and practice changing. The results provide a systematic evaluation of de-escalation of lymph node dissections in early-stage vulvar cancer. The first GROINSS-V study [28] was a multicenter observational study (from March 2000 until June 2006) investigating the safety and clinical utility of SLNBx using combination radioactive tracer and blue dye [29]. Patients with T1 VSCC < 4 cm in size that did not encroach on the vagina, urethra, or anus with DOI > 1 mm and clinically negative inguinal femoral nodes, underwent a wide local excision of the primary tumor in combination with a uni- or bilateral SLNBx. Standard H&E staining was used for pathologic assessment but ultrastaging was also performed if no metastases were initially identified. There was no further treatment if the patient was pathologically node negative (N0(sn)) but a full inguinofemoral lymph node dissection [IFND] was performed for node positive (N(+)) disease.

**Figure 2:**
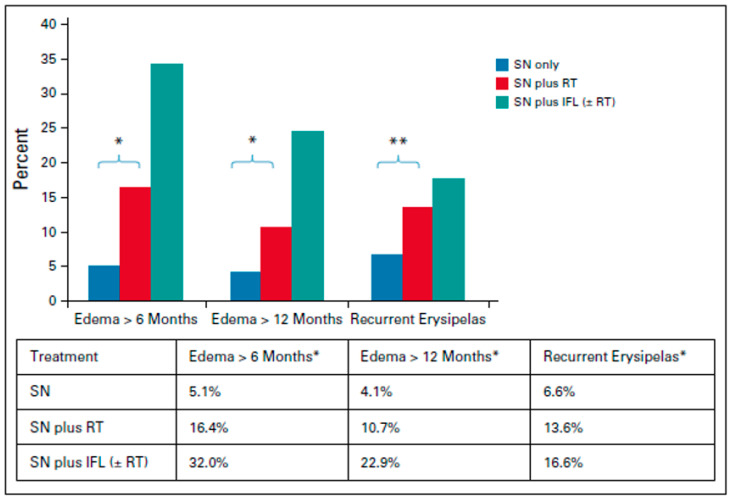
Long-term morbidity on GROINSS V-II/GOG 270. * *p* < 0.0001. ** *p* = 0.001. IFL, inguinofemoral lymphadenectomy; RT, radiation therapy. SN, sentinel node. Reprinted from Oonk et al, JCO 2021 [31].

Several important outcomes were concluded from GROINSS-V: (1) For early-stage vulvar-confined disease, SLNBx alone resulted in a low risk of relapse. Among the 623 negative SLNBx, only 2.3% groin recurrence rate was observed. This compared favorably to the 5% ipsilateral groin recurrence rate following negative full superficial inguinal dissection in a prospective GOG study of low-risk vulvar cancer [25]. (2) Patients who underwent a sentinel rather than a full IFND demonstrated lower groin wound breakdown (12% vs. 34%) compared to those who went on to a full inguinofemoral dissection following the sentinel procedure. [Table 2]. Long term follow-up of the GROINSS-V trial also demonstrated reduced rates of lymphedema from 1.9% in the SLNBx group versus 25% for those who had completed a full IFND. [Figure 2]. The 5- and 10-year recurrence rates for node-negative N0(sn) patients were 24.6% and 36.4% versus 33.2% and 46.4% in patients who had positive sentinel nodes with subsequent completion inguinofemoral node dissection [30]. (3) Although used to improve the detection of SLN metastases, through ultrastaging, they were able to capture lower nodal burden of disease and that in itself impacted disease-specific survival. Not surprisingly, they concluded that the risk of non-sentinel metastases increased with size of sentinel-node metastasis, but more importantly, (4) they identified that there was no size cutoff below which chances of a non-sentinel node metastasis risk was close to zero. Thus, establishing SLNBx alone to be an acceptable approach for N0 patients, but also confirming the necessity of adjuvant therapy in patients with (+) SLNBx.

### Adjuvant Radiotherapy for the Primary Tumor and Local Regional Lymph Nodes

Surgical margins of the primary lesion have been a classic indication for adjuvant therapy and a topic of debate in the modern literature. [Table 3]. Excision of 2 cm margins of grossly normal-appearing tissue has been recommended, when possible, with a goal of obtaining at least 1 cm microscopic margins in fresh tissue, and ≥8 mm following tissue processing. If the tumor involved or abutted the clitoris, preservation of this structure could not be possible.

The recommendation of ≥8 mm surgical margins is an established recommendation based on a retrospective series from UCLA and City of Hope demonstrating ~50% local recurrence rates with surgical resection margins < 8 mm [32]. Surgical margins, depth of invasion (DOI), tumor thickness > 10 mm, an infiltrative growth pattern, the presence of lymphovascular space invasion (LVSI), and increased keratin were all pathologic features that correlated with increased risk for local recurrence. In that particular series, 30% of patients with ≥10 mm DOI experienced a recurrence, in contrast to 0 of the 52 patients with a depth of stromal invasion < 2.5 mm.

Several publications have since gone to provide conflicting data as it relates to the 8mm cutoff. Faul et al. published a retrospective series evaluating the role of adjuvant radiation for close and positive surgical margins in which a 5mm margin of excision was a predictor for local recurrence [34]. Woebler and colleagues could not identify a significant effect on PFS based on margin distance when evaluated as a continuous variable or divided into groups (<3 mm, ≥3 to <8 mm, and ≥8 mm), nor did they observe a difference in local recurrence rates based on 8mm margin status (11.1% for margins < 8 mm and 10% for those ≥8 mm) [36]. A small single institutional series from University of Minnesota specifically evaluating Stage I patients with close or positive margins observed similar 2-year recurrence rates between patients who received no further therapy and those who underwent either margin re-excision or radiotherapy [37].

Additional modern series have put into question the relationship between invasive surgical margins and recurrence, with a 2019 series from the Netherlands suggesting that locoregional recurrence did not correlate with pathologic tumor free margins, but rather the presence of high-risk precursor lesions at the surgical margin such as lichen sclerosis, dVIN or both [38].

Current ESGO guidelines acknowledge that adequacy of surgical margin is variably defined in the literature with debate on the threshold for which adjuvant radiation is recommended [39]. Similarly, the NCCN guidelines acknowledge questions around 8 mm margins with some suggestion that smaller surgical margins are acceptable particularly in an effort to preserve sensitive areas and sexual function [19]. Despite these controversies, treatment for close surgical margin from pathologic tumor remains standard of care with an accepted radiotherapy dose range based on estimated risk [40]. In a retrospective review of 300 patients with Stage I–IVA vulvar cancer, Viswanathan and colleagues reviewed and scored pathologic slides based on margin resection status and evaluated corresponding rates of recurrence and local failures. In total there were 78 recurrences with 62 local failures. The 4-year rates of freedom from vulvar recurrence based on margin status were 82% for negative margins (defined as >1 cm after formalin fixation), 63% for close margins (<1 cm) and 37% for positive margins, respectively (*p* for trend = 0.005). On multivariate analysis, close margins (HR = 3.03, 95% CI 1.46–6.26) and positive margins (HR = 7.02, 95% CI 2.66–18.54) were associated with a significantly increased risk of vulvar relapse. Those who received a dose ≥ 56 Gy had a lower risk of relapse than those who received ≤ 50.4 Gy (*p* = 0.05) [35]. This relationship between dose and margin is often used in clinical practice and has been incorporated in more modern post-operative series such as the GROINSS V-III trial. Of note, in that particular series from Harvard, recurrences were noted with margins up to 9 mm, with the highest risk of vulvar recurrence associated with margins ≤ 5 mm (*p* = 0.002).

In 2016, a Consensus Statement was generated to ensure more uniform practices both for adjuvant and curative intent radiotherapy [22]. In addition to contouring guidance for radiation planning, adjuvant radiotherapy dose levels were recommended which encouraged a minimum biologically equivalent dose of 45–50 Gy be delivered to the operative bed for cases in which the surgical margins are clear, but acknowledged the need for dose escalation in response to close or positive margins or in the presence of LVSI. In general practice, most radiation oncologists will prescribe 56 Gy or higher dependent on the number of risk factors and concern for microscopic versus macroscopic residual disease.

With the recognition that any node-positive disease required an additional intervention, the GROINSS V-II (GOG 270) sought to determine the safety of inguinofemoral radiotherapy as an alternative to inguinofemoral lymphadenectomy in patients with vulvar cancer and a metastatic sentinel lymph node. On interim analysis it was determined that among patients with sentinel node micrometastases (≤2 mm), the ipsilateral isolated groin recurrence rate was 1.6% at 2 years with inguinofemoral radiotherapy. However, for those with sentinel node macrometastases (>2 mm) that were managed with 50 Gy radiotherapy, there was an unacceptably high rate of isolated groin recurrences at 22% with radiotherapy versus 6.9% with inguinofemoral lymphadenopathy. This data suggested that radiation could be a safe alternative to IFND with (+) micrometastatic SLNs < 2 mm, but also informed us that 50 Gy for SLN macrometastases > 2 mm was not a safe alternative to inguinofemoral dissection.

Drawing on the response rates seen in unresectable vulvar cancers with combination chemoradiation, the currently accruing GROINSS-VIII/NRG GY024 study is evaluating the safety of replacing inguinofemoral lymphadenectomy with the radiosensitizing-properties of concurrent chemotherapy along with radiation for early-stage vulvar cancer with sentinel node macrometastasis (>2 mm) and/or extracapsular extension (ECE). Initiated in 2021, research subjects with SN metastases > 2 mm and/or with extracapsular extension will be eligible for adjuvant chemoradiation with weekly cisplatin and a radiation (RT) dose of 56 Gy.

Currently, for those patients in which a SLNBx identifies a macrometastasis and/or ECE, the standard of care at this time remains a completion IFND, with the clearest indications for adjuvant radiation following IFND being >2 positive lymph nodes or >20% of submitted lymph nodes. This is supported by the results of GOG 37 [41] as well as the large retrospective exploratory multicenter AGO-CaRE-1 Study [42,43] that evaluated adjuvant therapy in lymph node positive vulvar cancer. Of note, although >2 positive lymph nodes was where the greatest benefit was seen, in modern clinical practice most radiation oncologists will offer radiotherapy for any lymph node involvement given the prognostic significance of LN+ disease as well as the observed smaller oncologic benefits seen in this population in both GOG 37 and AGO-CarE-1 studies. The recommendation for adjuvant radiation for any lymph node positive disease (>1 LN) is supported by the ESGO Guidelines [39] and included in the NCCN guidelines as well [19].

GOG-37 included operable patients who underwent vulvectomy and groin dissection. All lymph node positive patients were randomized intraoperatively to receive either RT to groin and pelvis versus pelvic lymph node dissection. Radiation to a total dose of 45–50 Gy was administered using older delivery techniques commonly used at that time, including a midline block to not overlap with the postop bed of the primary vulvectomy. The study determined that pelvic radiotherapy was associated with better oncologic outcomes when compared to pelvic node dissection in node positive (N+) patients, thus providing strong evidence for the use of radiation therapy in the management of vulvar cancer patients with positive groin lymph nodes and establishing it as a preferred treatment approach over pelvic node resection.

As a retrospective review of 1618 patients with stage IB-IVA vulvar cancer treated at centers in Germany, the AGO-CaRE-1 study [42] further supported the recommendation with observed improved 3yr PFS and OS outcomes with adjuvant radiotherapy for node-positive patients when compared to no adjuvant treatment. In that series the majority of N+ patients had one (172 [38.5%]) or two (102 [22.8%]) positive nodes with 244 (54.6%) N+ patients receiving adjuvant therapy. Radiotherapy fields consisted of radiation directed at the groins (±other fields)

In analysis of both GOG 37 and AGO-CaRE-1, several controversies remain as it relates to the impact of adjuvant therapy for patients with one positive lymph node. However, both series noted consistently observed decrement in oncologic outcomes for any nodal burden and at minimum suggested a possible benefit in those with nodal burden less than 2 nodes. Furthermore, in light of the GROINSS-VII data, we now have a greater understanding that the characteristics of that one lymph node may further delineate risk as well as any associated benefit from adjuvant therapy. A National Cancer Database [NCDB] series evaluating this clinical scenario also suggests benefit in this lower nodal burden population: Specifically, a 5yr disease-specific-survival [DSS] of 77% versus 61% in the setting of adjuvant radiotherapy for 1 positive lymph node. Radiation treatment was observed to improve the survival of those with less than 12 LNs removed in that publication [44].

Incorporation of adjuvant chemoradiation rather than radiation alone for lymph node positive disease developed largely in extrapolation from the cervix cancer literature with a limited number of publications suggesting a benefit for vulvar cancer patients. An NCDB study from Pittsburgh [45] evaluated the impact of adjuvant chemotherapy for node positive patients, suggesting a 38% reduction in death with the use of adjuvant chemoradiation for lymph node positive vulvar cancer. The majority of patients in that study had 1–3 involved lymph nodes (76.6%) with most patients (39.8%) having a single involved lymph node. A similar percentage of patients (39.4%) had lymph node positive ratios of >20%. A small series from Yale also demonstrated a trend in relapse-free survival [RFS] or PFS, DSS, and OS benefit of adjuvant chemoradiation over adjuvant radiation alone for high-risk vulvar cancer patients, but with limited statistical power due to the very small number of patients in their cohort [46].

In practice, adjuvant chemoradiation is often routinely utilized for >2 lymph nodes or extracapsular extension with less uniformity in practice as it relates to adjuvant radiation versus adjuvant chemoradiation for a single positive lymph node [47]. Current NCCN guidelines support the incorporation of concurrent chemotherapy for any level of nodal burden with Category 2A status in contrast to adjuvant radiation which carries a Category 1 recommendation.

## 3. Non-Operative Cancer of the Vulva

Surgical management alone for locally advanced disease yielded disappointing results and significant morbidity, which provided the foundation for interest in multimodality treatment around the mid to late 1980s [48,49]. Beginning in the late 1980s and throughout the 1990s and early 2000s, several publications demonstrated the use of neoadjuvant chemoradiation in an effort to facilitate resection with two landmark studies, GOG 101 [50] and GOG 205 [51] prospectively building on this experience.

GOG 101 was a prospective multi-institutional trial involving 73 unresectable Stage III/IV disease patients. Treatment consisted of a complex planned split course of concurrent cisplatin/5FU and 47.6 Gy radiation therapy followed by surgical excision of the residual primary tumor plus bilateral inguinal femoral LND. The course consisted of two 23.8 Gy treatment regimens delivered as a twice daily 1.7 Gy per fraction regimen for the first four days followed by daily 1.7 Gy per fraction for the remaining 10.2 Gy with an intentional 2-week break for treatment tolerability. Concurrent chemotherapy consisted of a constant Infusion of 5FUd 1000 mg/m^2^/day for days 1–4 of each split and Cisplatin 50 mg/m^2^ on day 1 of each split. The subsequent GOG 205 trial saw a more consolidated radiation treatment course with dose escalation to 57.6 Gy in 1.8 Gy fractions and weekly Cisplatin 40 mg/m^2^ for a maximum of 7 weekly cycles.

With each of these experiences there were increased rates of clinical complete response (cCR) and pathologic complete response (pCR): 48% and 31% in GOG 101 and 64% and 50% in GOG 205, suggesting a benefit to dose escalation in locally advanced vulvar cancer. [Table 4]. With the advent of intensity modulated radiotherapy [IMRT], modern-day radiotherapy practices have allowed for further dose escalation with most institutions prescribing 60–68 Gy to gross disease. Several single institutional retrospective experiences have demonstrated an even higher rate of clinical and pathologic complete response with an increased dose when compared to the historic rates seen in GOG 101 and GOG 205. The recently published Phase II GOG 279 trial [52] underscored the value of dose escalation with a 73% complete response rate with the use of IMRT with concurrent weekly cisplatin and gemcitabine. In GOG 279, 50–64 Gy was prescribed to the groin and low pelvis with 64 Gy to the vulva using IMRT + chemotherapy. Early clinical endpoints included 74% 12mo PFS and 70% 2-year OS, but also have opened discussions regarding escalation of therapy with the addition of gemcitabine, particularly for HPV independent disease.

There are several published series identifying that HPV or p16 positivity is associated with both higher rates of response to neoadjuvant or definitive chemoradiation [53] as well as overall better oncologic outcomes including PFS and fewer in-field relapses [54]. A meta-analysis of 33 studies and 7721 subjects concluded that HPV-positive disease was associated with better OS (HR = 0.64) and RFS or PFS (HR 0.66) compared with its HPV-negative counterparts [4].

Given normal tissue tolerances, escalation of therapy will most likely take the form of incorporation of additional systemic agents to compliment the benefits seen with radiotherapy.

## 4. Vulvar Cancer with Distant Metastatic Disease

For patients with recurrent or metastatic vulvar cancer, current treatment options are extremely limited with overall response rates to traditional platinum doublets ranging in the mid to low teens [55,56,57]. Radiation, when utilized, is palliative in nature.

In the modern era of immunotherapy (IO), a variety of IO agents have been tested in Ph I and 2 trials with some inclusion of vulvar cancer patients. The KEYNOTE-028 basket Ph Ib trial underscored the anti-tumor effects of pembrolizumab in diverse PD-L1 positive, advanced, solid tumors with a 6% objective response rate (ORR) seen in 18 vulvar cancer patients on that trial [58]. KEYNOTE-158 went on to evaluate pembrolizumab monotherapy in 101 metastatic vulvar cancer patients with prior treatment failure and demonstrated an objective response rate of 10.9% regardless of tumor PD-L1 expression with a median duration of response of 20.4 months in treatment responders [59].

Checkmate 358 evaluated the safety and efficacy of the immune check point inhibitor (ICI) Nivolumab, as monotherapy in recurrent or metastatic cervical, vaginal, or vulvar carcinoma. In a small cohort of 5 patients with recurrent and/or metastatic vulvovaginal cancers there was signal of a possible benefit with partial response in an HPV-negative vulvar cancer patient, and 12-month and 18-month OS rates of 40% (*n* = 2) and 20% (*n* = 1), respectively [60]. Lastly, another PD-1 ICI, Cemiplimab, has demonstrated an overall response rate of 47% for cutaneous advanced squamous cell carcinoma (SCC) in an open-label multicenter non-randomized Ph I/II study [61]. No primary vulvar cancers were included but vulvar SCC can be considered a cutaneous SCC and Cemiplimab has been included in treatment guidelines for advanced or metastatic disease with at least some signal in case reports to suggest clinical response with this agent [62]. This recommendation is in part because of the data seen in cutaneous SCCs but also due to the improved response rates seen with Cemiplimab in recurrent metastatic cervix cancer after first-line chemotherapy in the EMPOWER-CERVICAL-1 Ph 3 trial [63].

Outside of immunotherapy, targeted agents evaluating the use of EGFR inhibitors such as Erlotinib, VEGF-inhibitors such as Bevacizumab and Antibody–Drug Conjugates targeting HER-2 positive disease have also been reported.

In a Ph 2 study involving 41 patients divided into two cohorts (1) vulvar lesions amenable to surgery or chemoradiation or (2) those with metastatic measurable disease, short duration responses were observed with 67.5% deriving a clinical benefit with daily 150 mg Elotinib [12].

Combination bevacizumab and chemotherapy has been explored in both metastatic patients [64] and as a neoadjuvant regimen to facilitate surgical resection in locally advanced vulvar cases [65] with several case reports demonstrating impressive tumor regression [64].

ADCs targeting the HER2 receptor, which are prevalent in breast cancer management, have been of interest for all gynecologic cancers as our knowledge of Her2 positivity in gynecologic cancers advances. Less than 2% of vulvar SCCs are thought to be Her2 positive although 25% of vulvar extramammary Paget’s disease express positivity [66]. One vulvar patient was included in the DESTINY-PanTumor02 Ph 2 trial evaluating the ADC Trastuzumab deruxtecan, T-Dxd or EnHertu. There was a 30% ORR for the tumors in the ‘others’ category of that trial, which included the sole vulvar patient. On review, the patient experienced a duration of response of 2.6 months and PFS 5.6 months [67] Zanidatamab (ZW25) is a bispecific IgG1 monoclonal antibody targeting two domains on HER2. In a Ph I trial including a variety of gynecologic cancers (including endometrial, ovarian/fallopian and vulvar cancers), an ORR of 37% (31/83) was observed [68].

## 5. Future Directions in Clinical Research

### 5.1. Surgery

Current clinical trials are assessing the role of incorporating prognostic precursor information to inform surgical management. The currently accruing STRIVE trial [STRatIfication of Vulvar Squamous Cell Carcinoma by HPV and p53 Status to Guide Excision (STRIVE)] is a prospective study to determine if implementation of HPV (p16) and p53 stratified surgical margin management will improve outcomes. Patients with HPV-associated Vulvar Squamous Cell Carcinoma (VSCC) and <8 mm surgical margins regardless of in-situ (HSIL) margin status will be eligible for the de-escalation prospective study and those with HPV-independent VSCC and surgical margins < 8 mm or (+) dVIN and/or p53mutant on IHC will be randomized to re-excision versus observation. The hypothesis of the investigators is that de-escalation in the HPV-associated group will result in improved patient reported outcomes (PRO) without compromising oncologic outcomes, with evaluation of the benefit of margin re-excision to achieve clearer margins in the HPV-independent population [69].

### 5.2. Systemic Therapy

As has often been the case in vulvar cancer management, clinical care of cervix cancer has often laid the groundwork for subsequent innovation in vulvar cancer management given the commonality of HPV-driven disease. Similar to the trials in the 1980s and 1990s evaluating neoadjuvant chemotherapy prior to surgery in cervix cancer [70], the ongoing Phase II trial VULCANize (Treatment of Locally Advanced VULvar CArcinoma in a Neoadjuvant Setting With Carboplatin and Paclitaxel Chemotherapy) has been accruing with the goal of evaluating if response to neoadjuvant chemotherapy can allow for less extensive surgical resection [71]. Tistoumab-Vedotin or Tiv-Dak is an antibody drug conjudate (ADC) targeting tissue factor which has shown clinical benefits in cervix cancer with possible cross over in vulvar cancer and similarly, TROP2 inhibitors, particularly ADCs like sacituzumab govitecan, are being investigated for cervical cancer treatment and may be an actionable agent in vulvar cancer management as well.

In the non-operative setting there is recent Phase II data evaluating combination cisplatin with the PD-1 inhibitor pembrolizumab and radiation for patients with primary unresectable, incompletely resected, recurrent, or metastatic squamous cell carcinoma of the vulva. The radiation was definitive with median dose to the primary of 68.4 Gy and prophylactic 45 Gy to the pelvic, inguinal and vulva CTV. In this recently presented small series of 24 patients, predominantly unresectable patients (92%), the observed 6mo RFS/PFS was 70% and the median PFS had not been reached at time of presentation. Of note, PD-L1 was positive in all patients and there was an increase in mean TCR clonality after 2 cycles [72].

There are additional trials that are in development or ongoing in both the US and in Europe including the Apollo trial, a prospective multicenter phase II non-controlled clinical trial in 40 VSCC patients who will receive neoadjuvant Pembrolizumab prior to anticipated surgical resection. In the recurrent and metastatic setting there is an ongoing Ph II study evaluating the safety and efficacy of an anti PD-1 and CTLA-4 bispecific antibody (AK104), and the PIERCE trial evaluating Pembrolizumab in Combination with Lenvatinib in patients With Recurrent, Persistent, Metastatic or Locally Advanced Vulvar Cancer Not Amenable to Curative Surgery or Radiotherapy.

Next generation sequencing has also identified a number of potential actionable mutations for systemic therapy, unveiling a deeper understanding of the genetic makeup of vulvar cancers. There are ongoing clinical trials and developing concepts evaluating a variety of molecular pathways including PI3K, AKT and mTOR pathways.

### 5.3. Radiotherapy

With the advent of intensity modulated radiotherapy [IMRT] management for this disease site was revolutionized in its ability to not only dose escalate on disease but also spare normal tissues. Despite technological advancement, vulvar cancer radiotherapy continues to be a challenging treatment course due to the involved anatomy and nearby functional organs. Population-based studies [73] report that only 51% of patients receiving external beam for vulvar cancer were able to receive treatment and meet known optimal RT delivery metrics including >20 treatment fractions, overall treatment time (OTT) < 8 weeks, and <1-week treatment break. Compliance to optimal radiotherapy delivery metrics is known to result in improved overall survival [73,74,75] but can be challenging for patients due to known and expected sequalae of radiotherapy.

For better tolerance of combined modality therapy, delivering upfront boost followed by pelvic field external beam may help improve compliance to optimal radiotherapy delivery metrics. Chadha et al. have reported early results on 4 elderly (>70 years) patients with locally advanced vulvar cancer treated with concomitant cisplatin and external beam radiotherapy [76]. All patients received >30 EBRT fractions, treatments averaged over 58 elapsed days [range: 44 days to 69 days] approaching the optimal metric of 8 weeks, and no patients had ≥7 days [ranged from 4 days to 6 days] intra-treatment break. Furthermore, all were able to receive at least 5 weekly cisplatin doses. Similar experience from the Dutch phase II study [77] on definitive chemoradiation that used a flipped sequence with upfront boost followed by elective pelvic field without scheduled treatment break was noted. They reported high rates (90%) of compliance in completing radiotherapy per protocol. Such modifications of fractionation schedules, especially when delivering concomitant chemotherapy may be considered for improved compliance to achieving optimal protocol treatment metrics.

As it relates to palliation, data from the ELECHTRA (ELEctroCHemoTherapy vulvaR cAncer) Study evaluated the use of electrochemotherapy with bleomycin for recurrent vulvar cancer with encouraging quality of life results [78].

## 6. Summary

Vulvar cancer is a rare complex malignancy for which we continue to develop both our risk-reducing interventions and improve clinical outcomes. As we grow in our understanding of the various molecular subtypes of this disease, the triaging of therapies based on established molecular prognostic indices and risk-tailored treatments will help to guide management.

## Figures and Tables

**Table 1:** Histopathologic patterns of vulvar squamous carcinoma (85% of vulvar cancer lesions).

Factor	Keratinizing Squamous Carcinomas	Basaloid Squamous Carcinomas
Prevalence	80%	20%
Age	Older	Younger
Related Disease	LS and other vulvar dystrophy	HPV infection, other anogenital lesions, VIN, multifocality
p16	often (−)	often (+)
p53	either (+) or (−)	Often (−)

**Table 2:** Surgical Complication Rates in GROINSS V-I. Data from Van der Zee et al, JCO, 2008 [29] and Te Grootenhuis et al, Gyn Onc, 2016 [30].

Side-Effects	IFN Dissection	SLN Surgery	
Wound breakdown	34%	11.7%	*p* < 0.0001
Cellulitis	21.3%	4.5%	*p* < 0.0001
Lymphedema	25.2%	1.9%	*p* < 0.0001

IFN: Inguinofemoral node dissection; SLN: sentinel lymph node surgery.

**Table 3:** Observed relationship of Surgical Margins status and Local Recurrence Risk.

	UCLA/CoH Heaps et al. [32]	Irvine Chan et al. [33]	Pittsburgh Faul et al. [34]	BWH/DFCI Viswanathan et al. [35]
Study period	1957–1985	1984–2000	1980–2004	1980–2009
No: of patients	135	90	62	205
No: of patients with close/positive	44	60	62	116
Close margin	<8 mm	<8 mm	<8 mm	<5 mm
% patients receiving RT	0%	20%	50%	30%
Local recurrence	47.7%	23%	58% no RT 16% with RT	38.9%

**Table 4:** Prospective chemoradiation trials for locally advanced vulvar cancer demonstrating improved response rates with radiotherapy dose escalation.

	GOG 101 [50] Phase II	GOG 205 [51] Phase II	GOG 279 [52] Phase II
Protocol	2 cycles of 5FU + cisplatin 47.6 Gy × 1.7 G fx. Split course RT with break Biopsy/Surgery 4–8 weeks later	Weekly cisplatin 45 Gy with boost 57.6 Gy to gross disease Biopsy/Surgery 4–8 weeks later	Weekly cisplatin + gemcitabine IMRT 45 Gy with boost 64 Gy to gross primary/nodes Imaging evaluation 4–6 weeks with FNA to confirm path status
Evaluable # of patients	71	58	52
Clinical complete response (CCR)	34 (48%)	37 (64%)	37 (71%)
Complete pathologic response (CPR)	22 (31%)	29 (50%)	38 (73%)
